# Outcome Evaluation in Social Comparison: When You Deviate from Others

**DOI:** 10.3390/brainsci13060925

**Published:** 2023-06-07

**Authors:** Shinan Sun, Yang Wang, Xuejun Bai

**Affiliations:** 1 Faculty of Psychology, Tianjin Normal University, Tianjin 300387, China; 2 Key Research Base of Humanities and Social Sciences of the Ministry of Education, Academy of Psychology and Behavior, Tianjin Normal University, Tianjin 300387, China

**Keywords:** social comparison, outcome evaluation, deviation degree, electroencephalogram (EEG), event-related potential (ERP), FRN, P300

## Abstract

Individuals often measure their performance through social comparison. With the increase in the deviation degree between the self and others, the outcome evaluation of individuals’ abilities in the social comparison context is still unknown. In the current study, we used a two self-outcomes × three others’ outcomes within-participant design to investigate the effect of the deviation degree of the self versus others in the social comparison context. Event-related potentials (ERPs) were measured while participants performed a three-person dot estimation task with two other people. When participants received positive results, the amplitudes of feedback-related negativity (FRN) and P300 showed a significant gradient change in the degree of deviation between the self and others (even win vs. better win vs. best win conditions). However, we did not find a similar progressive effect when participants received negative results (even loss vs. worse loss vs. worst loss conditions). These findings suggest that the deviation degree affects the primary and later processing stages of social comparison outcomes only when individuals received positive outcomes, which may reflect how people develop an empathic response to others. In contrast, people tended to avoid deeper social comparison that threatened their self-esteem when they received negative outcomes.

## 1. Introduction

In the real world, people rarely have absolute standards for processing information. Hence, people often measure their performance relative to that of others through social comparison [1]. The dimension of comparison can be anything relevant to the individual, for example, abilities, morals, or fortune [2,3,4]. Ability is an important social comparison dimension with a high degree of self-relevance. Social comparisons of ability entail comparisons of task performance and individual achievement [5]. A common example is when students assess their academic performance by comparing their grades with those of their peers. In this age of fierce competition, individuals indulge in social comparison to others even in noncompetitive contexts [6].

Previous studies have assessed how social comparisons are supported by various networks, such as the empathic system and the reward system [7,8]. Empathy is typically conceptualized as a positive emotion that expresses love and help [9,10]. The precuneus and temporo-parietal areas are often thought to be associated with empathy and mentalization, and are used when individuals need to understand and predict other people’s intentions and beliefs [11,12,13]. Instead of feeling threatened when they perform poorly, individuals are more empathetic when they perform well [14]. However, when they perform worse than others in social comparison, even prosocial people express less sympathy for the failure of disliked players [15]. The ventral striatum (VS), medial prefrontal cortex (mPFC), and dorsal anterior cingulate cortex (dACC) constitute the main structures of the reward system. VS activity depends on relative rewards [16]. In a social comparison context, what caused VS activity to increase was earning more than others. In addition, the relative information represented in the VS can be used for an individual’s future decision making and predict mPFC activity in a subsequent trial [17]. Previous research has shown that individuals adjust their behavior based on the performance of others in order to obtain higher rewards [17]. Thus, the VS and mPFC network encodes the subjective value of the expected reward [18]. The dACC is a region that plays a key role in prediction errors [7]. For example, when individuals make upward social comparisons, they report stronger feelings of jealousy and show increased activation in the dACC [19]. Such error signals produced by the dACC can also be measured as FRN in electrophysiology [20]. We will expand on this in a later paragraph.

EEG has been used to access brain function in healthy and pathological individuals due to its high temporal accuracy [21,22]. Using the ERP technique, researchers have examined the components associated with the outcome processing of social comparison. Based on previous studies, the FRN and P300 components are highly relevant to individuals’ processing of social comparison outcomes [15,23]. The FRN component is a negative wave that reflects the individual’s expected error and peaks approximately 200–350 ms after the stimulus presentation [24]. In social comparison, unexpected results can induce a more negative-trending FRN amplitude [25]. For instance, others’ gain elicited a larger FRN than others’ loss in the self-loss condition (i.e., monetary gain or loss). The P300 component is a positive wave that reflects the deep attentional processing of stimulus information and peaks approximately 300–500 ms after the stimulus presentation [26]. In social comparison, the P300 component is related to an individual’s motivational and affective evaluation [27,28]. Positive results can induce a more positive-trending P300 amplitude [23].

Several ERP studies have endeavored to elucidate the time course of social comparison between individuals (i.e., with only one comparison target). For instance, Zhang [29] asked participants to play a two-person gambling game repeatedly with a same-sex friend or a stranger. The ERP results showed that in the primary stage (as indexed by the FRN) of outcome processing of social comparison, the FRN was more negative for others’ gain than for others’ loss in the self-loss condition. This finding suggests that we spontaneously make various social comparisons and that outcome evaluations are influenced by social comparisons. Wu [2] used a dot estimation task to prime the absolute results (gain or loss) and the relative monetary reward ratio (1:1, 1:2, or 2:1) of each. The ERP results showed that positive outcomes and higher rewards induce larger P300 amplitudes. Their finding suggests that the late stage (as indexed by the P300) of outcome processing is sensitive to the valence and magnitude of outcomes.

A considerable number of ERP studies have investigated the time course of social comparison between individuals. However, with the increase in the deviation degree between the self and the group (i.e., if the comparison targets increase to two or more), the outcome evaluation of individuals’ abilities in the social comparison context is still unknown. In modern society, we make social comparisons not only with a single target but also with a group or even with strangers in our daily life [30]. The current educational system also provides invisible standards of comparison that encourage students to perform better in their groups. To our knowledge, only a few studies have explored outcome evaluation in social comparison through a three-person gambling experimental paradigm [15,31]. The gambling task reflects the individual’s level of luck, especially gambling luck, which may indicate the gambler’s low self-relevance in life. In contrast, ability is an important social comparison dimension with a high degree of self-relevance [32]. A functional magnetic resonance imaging study showed higher increased activation in the orbital frontal cortex and striatum when individuals process comparative outcomes in the skill game than when they play a luck game [33]. In addition, Festinger [1] reported that individuals are particularly inclined to evaluate their abilities and opinions through social comparisons. Researching the deviation degree between the self and the group in an ability context could help further elucidate the underlying physiology mechanism of social comparison.

In this study, we aimed to investigate the outcome processing of social comparison between the self and others, concerning different deviation degrees, in the ability context. In the current competitive society, people can make social comparisons not only with a specific person but also with a group. For example, previous studies have found that individuals compare themselves with their groups and tend to subjectively rate themselves as better than average [34,35]. When individuals make upward social comparisons, they may feel their self-esteem and social status are threatened by poor performance, which may then motivate them to do better. In contrast, downward social comparisons may not only restore an individual’s self-esteem and induce a sense of superiority but may also lead individuals to develop an empathic response toward others [14,36]. For both the upward and downward directions, it is still unknown whether individuals tend to engage in or avoid social comparisons when their performance gradually deviates from that of others in the ability context. The investigation of this issue has important practical significance in the field of social mentality and individual mental health, which can also improve the current theories of social comparison. For this purpose, we used a dot estimation task to explore social comparison as elicited in a context of a simulated ability test. The dot estimation task requires participants to estimate the number of dots randomly distributed on the screen in a short period [16]. This task reflects the individual’s spatial response estimation ability and is, therefore, widely used by researchers to create a social comparison context [37,38].

For the ERP outcomes, we hypothesized that the effect of the deviation degree of the self and others in social comparison would be reflected in the FRN and P300 components. We also hypothesized that when participants received positive results, the FRN and P300 amplitudes would show progressive changes with the degree of deviation between the self and others. Specifically, the amplitude of FRN increased significantly with increasing levels of self-versus-others differences; the amplitude of P300 decreased significantly with such differences. However, participants’ self-evaluation was threatened when they received negative results. At this point, individuals might tend to avoid further upward social comparison. Thus, when individuals perform poorly, the FRN and P300 amplitudes would not show progressive changes with the degree of deviation between the self and others.

## 2. Materials and Methods

### 2.1. Participants

We recruited 35 undergraduate and graduate participants (19 females) aged between 18 and 24 years (*M*age = 20.37 years, *SD* = 2.17) from Tianjin Normal University, China. Data from three participants (one female and two males) were excluded from the analysis due to excessive body movement. Therefore, 32 participants (18 females) were ultimately analyzed (*M*age = 20.31 years, *SD* = 2.16). All participants were right-handed, heterosexual, and had no history of psychiatric or neurological disorders. All of them reported normal or normal and/or corrected vision. Before entering the EEG laboratory, written informed consent was obtained from all participants. At the end of the experiment, each participant was given a base payment of CNY 60 for participation and a bonus of up to CNY 10 based on their performance in the task. The study protocol was approved by the Research Ethics Review Board of Tianjin Normal University.

### 2.2. Measures

**Figure 1:**
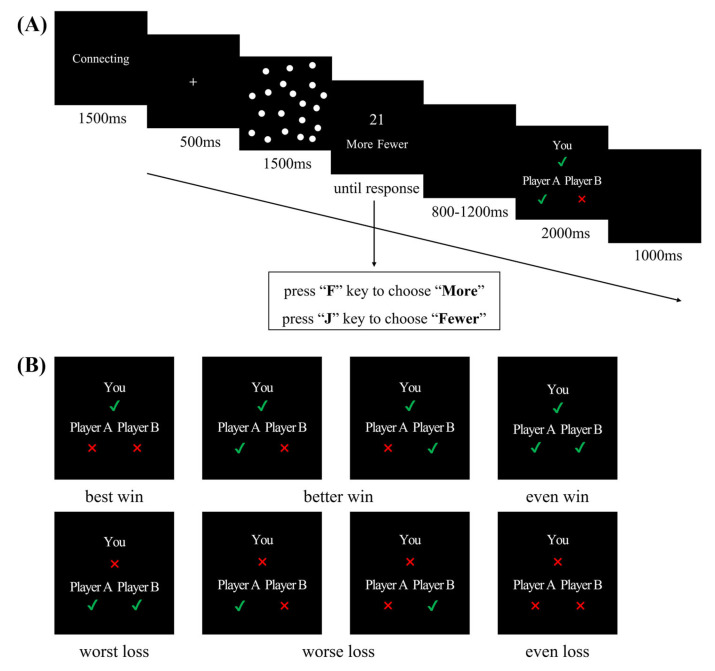
(**A**) Schematic representation of the three-person dot estimation task. (**B**) Eight types of result feedback in the dot estimation task.

Based on previous studies, we used a modified three-person dot estimation task as the ability task [38,39]. As illustrated in Figure 1, at the beginning of each block, a 1500 ms “connecting” was first presented. During the task, each trial began with a white fixation presented on a black background for 500 ms. Next, a varying number of 20 to 34 white dots were displayed on the screen for 1500 ms. When an integer appeared on the screen, the participants had to decide as quickly and accurately as possible whether they were seeing more or fewer dots than the presented integer. They were asked to press the “F” key on the keyboard with the left index finger to indicate that there were more dots than the presented integer and to press the “J” key with the right index finger if they thought that there were fewer dots than the presented integer. After an interval of 800 to 1200 ms, the outcomes for themselves and the other two players were displayed in a triangular distribution for 2000 ms. Participants were informed that a green “√” or red “×” would be displayed on the screen to indicate the correct or incorrect answers.

Unbeknownst to the actual participants, the other two people on the task were experimental assistants who would not be taking part in the actual experiment. Therefore, only one participant performed in the experiment and recorded the EEG data. In addition, the difference between the number and the dots presented earlier was only ±1. That is, the accuracy of the participants in the task tends toward the chance level. Based on this manipulation, their relative outcomes were predetermined by a computer program. Each type of outcome was presented to the participants the same number of times without arousing suspicion about the experimental process [2,38]. To eliminate the interference of the practice effect, the experimental material consisted of 60 dot images created by MATLAB 2021a. With this manipulation, each image appeared only 8 times out of a total of 480 trials. For example, first, an image with 20 dots appeared on the screen; next, the number 19 or 21 appeared four times each on the next screen.

### 2.3. Experimental Design

The experiment had a 2 self-outcomes × 3 others’ outcomes within-participant design. In the result feedback screen, the relative outcomes for the self and others were labeled as one of three conditions when the participant solved the task correctly: best win (others: both incorrect, 60 trials), better win (others: one correct and one incorrect, 120 trials), even win (others: both correct, 60 trials). Similarly, the relative outcomes were labeled as one of three conditions when the participant solved the task incorrectly, specifically worst loss (others: both correct, 60 trials), worse loss (others: one correct and one incorrect, 120 trials), and even loss (others: both incorrect, 60 trials).

### 2.4. Procedure

The participants were asked to come to the EEG laboratory to take part in an ability test simultaneously with two other anonymous players of the same gender from the same university. Before the experiment, the researchers led the three people into three separate rooms and informed each that the computers in the three rooms would be connected via a local area network. Unbeknownst to the participant, the two other people in the task were experimental assistants who would not be taking part in the actual experiment. Therefore, only one participant performed the task in the experiment and had their EEG data recorded during the dot estimation task.

Before the formal experiment, participants received the experimental instructions and practiced the dot estimation task for eight trials. The participants were told to do their best to complete the task, which would determine the bonus they eventually received. During the task, the participants were seated in an electrically shielded room with their eyes approximately 75 cm from the computer monitor. The stimulus presentation of the experimental task and behavioral data acquisition were controlled using E-Prime 3.0 software (PST, Inc., Pittsburgh, PA, USA) on a computer monitor with 1024 × 768 pixels resolution and a 60 Hz screen refresh rate. The dot estimation task consisted of 12 blocks with 480 trials. The number of trials for each condition was counterbalanced between the blocks. The participants were allowed to rest between blocks. Overall, the experiment lasted about 70 min.

At the end of the completed experiment, participants were asked whether the dot estimation task reflected their relative ability compared to others and the credibility of the scenario. All participants indicated that the dot estimation task reflected the relative ability between individuals and had no doubts about the experiment setup.

### 2.5. EEG Recording and Analysis

The EEG was recorded from a 64-channel Ag/AgCl electrode cap (NeuroScan, Melbourne, VIC, Australia) according to the international 10–20 system. Vertical electrooculograms were recorded, with two electrodes placed above and below the left eye. Horizontal electrooculogram recording electrodes were placed 1.5 cm to the outer canthi of both eyes. The reference electrode was placed in FCz and re-referenced offline to the average of the left and right mastoids. During the task, the impedance of all recording electrodes was kept below 5 kΩ. The EEG data were amplified with a bandpass filter of 0.05–400 Hz and sampled at 1000 Hz.

The EEG data were analyzed offline using the software CURRY 8. The data were filtered with a low pass of 30 Hz (24 dB/octave). The independent components analysis (ICA) method was used to remove the ocular artifacts. The continuous EEG data were extracted into epochs from −200 ms to 600 ms after the feedback screen onset. Separate epochs were corrected at the baseline with an interval of a −200 to 0 ms window before the feedback presentation. To exclude epochs contaminated by artifacts, epochs with voltages exceeding ±100 μV in any of the channels were excluded from further analysis. After the offline analysis, the average number of remaining trials was 47 (even win condition, a total of 60 trials), 95 (better win condition, a total of 120 trials), 48 (best win condition, a total of 60 trials), 47 (worst loss condition, a total of 60 trials), 93 (worse loss condition, a total of 120 trials), and 47 (even loss condition, a total of 60 trials), respectively.

According to the grand-averaged ERPs and previous studies [15,29,31], we focused on the FRN and P300 components. Specifically, we calculated the mean amplitudes within the time window of 240–300 ms for the FRN component across nine electrode locations (Fz, F3, F4, FCz, FC3, FC4, Cz, C3, and C4), and the time window of 310–410 ms for the P300 component across nine electrode locations (Cz, C3, C4, CPz, CP3, CP4, Pz, P3, and P4) following the feedback presentation. The results of descriptive statistical analysis showed that the Fz had the largest FRN mean amplitudes and the CPz had the largest P300 mean amplitudes. Therefore, in the results section, we focus on the analyses of the Fz and CPz electrodes.

Behavioral and ERP data were statistically analyzed using SPSS software version 25.0. The FRN and P300 amplitude were analyzed using a two-way repeated measures ANOVA of 2 self-outcomes × 3 others’ outcomes in a three-person social comparison. The significance level was set at 0.05 for all analyses. The Greenhouse–Geisser correction was conducted to account for sphericity violations whenever appropriate. Post hoc testing of the significant main effects was applied using Bonferroni adjustments. The partial eta-squared (η^2^_p_) values are provided to demonstrate effect size in ANOVAs.

## 3. Results

### 3.1. Behavioral Results

**Figure 2:**
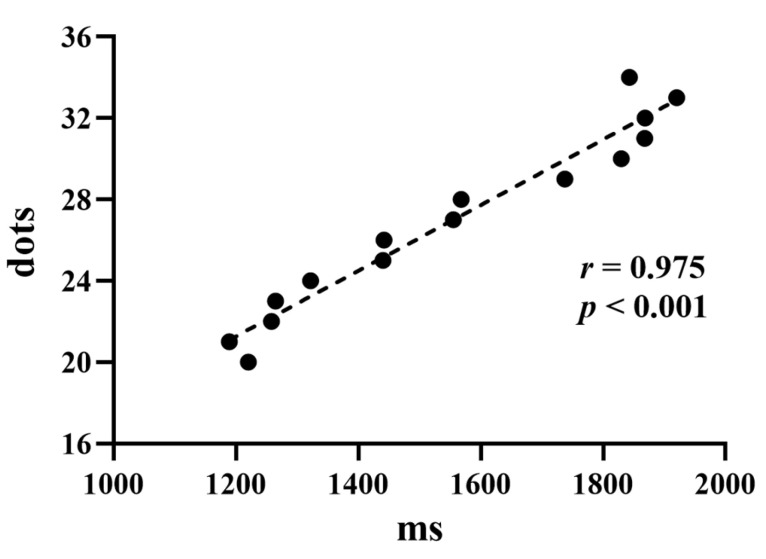
Scatter graph showing the correlation between participants’ mean RT and a varying number of 20 to 34 white dots.

During the dot estimation task, the average response accuracy of participants was 51.09% ± 0.64% (*M* ± *SE*). The one-sample t-test results showed that there was no significant difference between participants’ response accuracy and the chance level, *t*(31) = 1.703, *p* = 0.099. This result shows that our experimental manipulation of participants’ actual accuracy rate was effective. Therefore, participants had no doubts about the experiment setup’s judgment feedback, which was also consistent with participants’ subjective reports. In addition, participants’ average reaction time (RT) for decision making was 1555.12 ± 270.04 ms. We calculated the Spearman correlation and found a positive correlation between participants’ average keystroke RT and the number of dots displayed on the screen during the dot estimation task (*r* = 0.975, *p* < 0.001). This result indicated that participants spent more time completing the task as the number of white dots increased on the screen (Figure 2). It also provided evidence of how engaged and conscientious the participants were in the task.

### 3.2. ERP Results

#### 3.2.1. The FRN Component

**Figure 3:**
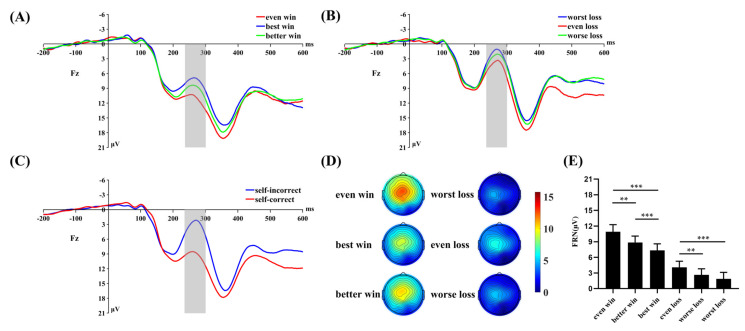
(**A**−**C**) Grand average ERP waveforms at the electrode site of Fz. The gray areas highlight the time window of the FRN (240−300 ms) used for statistical analysis. (**D**) The scalp topographic distributions of the FRN for each condition. (**E**) Bar graph showing the mean value of the FRN amplitude for each condition. Error bars indicate the standard errors. ** *p* < 0.01, *** *p* < 0.001.

For the FRN amplitude, repeated measures ANOVA (Figure 3) revealed a significant main effect of self-outcome, *F*(1, 31) = 151.986, *p* < 0.001, η^2^_p_ = 0.831. The FRN was larger for the self-incorrect (2.87 ± 1.17 μV) than for the self-correct (9.03 ± 1.26 μV). Meanwhile, the main effect of others’ outcome was significant, *F*(2, 62) = 3.469, *p* = 0.037, η^2^_p_ = 0.101. However, post hoc tests revealed no significant differences between any of the conditions (others-both correct, 6.39 ± 1.25 μV; others-one correct and one incorrect, 5.76 ± 1.15 μV; others: both incorrect, 5.70 ± 1.21 μV; *ps* > 0.05). Furthermore, we found a significant interaction effect of the self-outcome with others’ outcome, *F*(2, 62) = 43.842, *p* < 0.001, η^2^*_p_* = 0.586. A simple effect analysis revealed that when participants received positive results, the FRN was larger for the best win condition (7.33 ± 1.28 μV) than for the better win (8.86 ± 1.22 μV, *p* < 0.001) and even win condition (10.91 ± 1.36 μV, *p* < 0.001). The difference between the better win and even win conditions was also significant (*p* = 0.005). When participants received negative results, the FRN was larger for the worst loss (1.88 ± 1.24 μV, *p* < 0.001) and worse loss condition (2.65 ± 1.15 μV, *p* = 0.001) than for the even loss condition (4.07 ± 1.19 μV). The difference between the worst loss and worse loss condition was not significant (*p* = 0.174).

#### 3.2.2. The P300 Component

**Figure 4:**
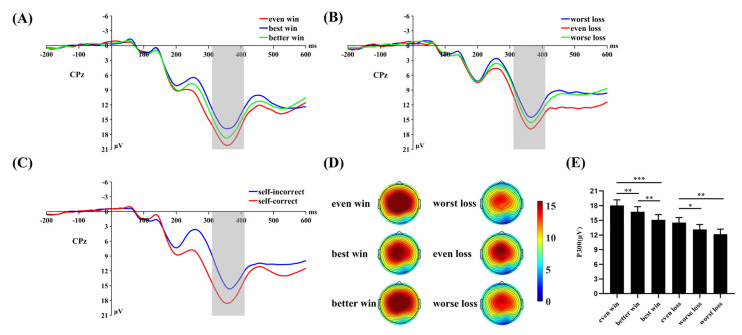
(**A**−**C**) Grand average ERP waveforms at the electrode site of CPz. The gray areas highlight the time window of the P300 (310−410 ms) used for statistical analysis. (**D**) The scalp topographic distributions of the P300 for each condition. (**E**) Bar graph showing the mean value of the P300 amplitude for each condition. Error bars indicate the standard errors. * *p* < 0.05, ** *p* < 0.01, *** *p* < 0.001.

For the P300 amplitude, repeated measures ANOVA (Figure 4) revealed a significant main effect of self-outcome, *F*(1, 31) = 30.837, *p* < 0.001, η^2^_p_ = 0.499. The P300 was larger in the self-correct (16.64 ± 1.06 μV) than in the self-incorrect (13.29 ± 0.97 μV) condition. Meanwhile, the main effect of others’ outcome was not significant, *F*(2, 62) = 0.385, *p* = 0.682, η^2^_p_ = 0.012. Furthermore, the interaction of self-outcome with others’ outcome was significant, *F*(2, 62) = 38.243, *p* < 0.001, η^2^_p_ = 0.552. A simple effect analysis revealed that when participants received positive results, the P300 was larger for the even win condition (18.05 ± 1.14 μV) than for the better win (16.75 ± 1.03 μV, *p* = 0.005) and best win conditions (15.11 ± 1.07 μV, *p* < 0.001). The difference between the better win and best win conditions was also significant (*p* = 0.002). When participants received negative results, the P300 was larger for the even loss condition (14.53 ± 1.06 μV) than for the worse loss (13.15 ± 1.01 μV, *p* = 0.031) and worst loss conditions (12.18 ± 1.02 μV, *p* = 0.001). The difference between the worse loss and worst loss conditions was not significant (*p* = 0.087).

## 4. Discussion

In the present study, we examined outcome processing in the social comparison context, from the perspective of the deviation degree, among the self and group members. The effect of the deviation degree of the self versus others in the social comparison was reflected in the FRN and P300 components of the ERP results. Specifically, this effect occurred when participants received positive outcomes rather than negative outcomes. Overall, when we make social comparisons with others, the deviation degree plays an important role in both the early and late stages of outcome processing.

### 4.1. The Deviation Degree Affects the Primary Processing Stage of Social Comparison Outcomes

For the time course of social comparison, the FRN component reflects the individual’s expected error [40]. Unexpected negative results could induce a larger FRN [41]. Similarly, positive feedback that exceeds participants’ expectations can induce a larger FRN [42]. In the current study, we first replicated classic findings from previous studies—that the main effect of self-outcome was significant in the early processing stage of social comparison outcomes [14,43]. The FRN component reflects the outcome against individual expectations [24]. In our study, the outcomes of the comparison reflect the ability of the individuals involved in the task. In addition, people tend to maintain positive self-evaluations [32]. Being disadvantaged by poor performance is usually not in line with our expectations. Thus, we observed a significant main effect of self-outcome. Specifically, the FRN was larger for the self-incorrect than for the self-correct condition.

For the FRN component, we found a significant interaction effect of the self-outcome with others’ outcome. When participants received positive results, the FRN amplitude showed a significant gradient change for the deviation degree between the self and others. More specifically, the largest FRN amplitudes were induced in the best win condition and relatively small FRN amplitudes were induced in the even win condition. This is consistent with the findings of Luo [31]. In our social comparison context, the FRN amplitudes induced by the different conditions were larger than the even conditions, regardless of whether participants received positive or negative outcomes. The difference between the best win and better win condition was also significant. Previous studies have shown that societies in collectivistic cultures place more emphasis on collectivist values and membership within groups [44,45]. Instead of feeling threatened when they perform poorly, individuals are more empathetic when they perform well [14]. Empathy is typically conceptualized as a positive emotion that expresses love and help [9,10]. A two-person social comparison study with Chinese participants by Sun [38] also found that the FRN was larger for others: incorrect than for others: correct in the self-correct condition. People align themselves with others in social situations, which can meet the criteria for good group membership [46]. Based on these findings, we suggest that when people received positive results, any deviation may be detected as an expected error, thus inducing a larger FRN.

When the participants received negative results, we did not find a similar effect. Both the worst loss and worse loss conditions induced significantly larger FRN amplitudes than the even loss condition. However, the difference between the worst loss and worse loss condition was not significant. First, in the social comparison context, one’s relative inferiority may have led to a threat to self-esteem, which induced a larger FRN [29]. Therefore, when individuals received negative results, the FRN induced by the even loss condition was significantly smaller than the FRN induced by other conditions. Bault [47] found that in the evaluation of social outcomes, the weight of gain is greater than that of loss. When individuals are directly involved in a task, they are primarily concerned with their winning or not [48]. On this basis, to maintain a positive self-concept and avoid further threats to self-esteem, people tend to withdraw from processing relative outcomes and avoid deeper upward social comparison [49,50]. Therefore, the difference between the FRN induced by the worst loss and the worse loss condition was not significant.

### 4.2. The Deviation Degree Affects the Later Processing Stage of Social Comparison Outcomes

Following the FRN, the P300 component of outcome processing in social comparison is generally considered to be related to individuals’ sustained attention and motivational or affective coding [27,28]. In the current study, we replicated the classical results of previous studies [51]. We found that the P300 induced by the self-correct condition was significantly larger than the P300 induced by the self-incorrect condition. This result reflects that individuals have a stronger motivation to perform well. 

Notably, we found that the interaction of the self-outcome with others’ outcome was significant. Consistent with the results of the FRN component, when the participants received positive results, the amplitude of P300 varied significantly with the degree of difference between the self and others. The P300 was larger for the even win condition than for the better win and the best win conditions. The difference between the better win and best win conditions was also significant. In the later advanced processing stage of social comparison, participants focus on the social significance of the relative outcomes [31]. In our study, the best win condition reflects that individuals run counter to others and have a potential interpersonal conflict problem. Conversely, the even win condition reflecting the reconciliation of this conflict has the greatest motivational significance and, thus, induces the largest P300 amplitude. In addition, Wu [2] found that the P300 is sensitive to the valence and relative amounts of outcomes. Therefore, another possible explanation is that the positive outcomes feedback has positive valence and there are different numbers of positive outcomes among the three social comparison conditions. The even win condition, reflecting the maximization of common interests, induces the largest P300 amplitude. 

When participants received negative results, the worst loss and worse loss conditions induced significantly smaller P300 amplitudes than the even loss condition. However, the difference between the worst loss and worse loss conditions was not significant. The reason the P300 was not sensitive to the degree of deviation when participants received negative results could be that participants tend not to allocate attentional resources to others for social comparison at the time [49,50]. Comparing themselves to others may threaten participants’ positive self-evaluation and they may experience the pain of performing worse than others. Hence, we did not find a significant difference between the worst loss and worse loss conditions.

In the later processing stage of social comparison, outcomes with differences may lead to stimulation processing with stronger motivational relevance and higher levels of autonomic arousal [52,53,54]. This suggests that timely and effective responses are needed in real life to reconcile potential conflicts between oneself and others [31]. Specifically, when we perform poorly, we can prevent threats to self-esteem by avoiding further excessive upward social comparisons. When we have an advantage, we can maintain good social relationships by showing concern for others in the group.

Human judgment is inherently comparative. In today’s society, people have more and more access to various social information, which has made social comparison targets expand to a wider range. Many traditional theories predict the direction and outcome of social comparison. Collins proposed in his construal theory that upward comparison leads to self-improvement and can be interpreted as showing similarity to the better-off target [55]. Tesser’s self-evaluation maintenance model (SEM) focuses on the level of intimacy with the comparison targets [56]. According to this theory, we may be proud of our in-group members when they do well, but we may also feel inferior when their performance dwarfs ours. Our study further refines the existing theory. That is, for both the upward and downward directions, individuals tend to engage in or avoid social comparison when their performance gradually deviates from that of others. We suggest that individuals tend to align themselves with others in the group in social comparison when they receive positive outcomes. However, when individuals receive negative outcomes, in order to maintain a positive self-concept and avoid further threats to self-esteem, people tend to withdraw from processing relative outcomes and avoid deeper upward social comparison. 

### 4.3. Limitations

The current study has some limitations. First, the social comparison participants in our study were all college students from the same university. Follow-up studies should also focus on the group identification of the social comparison targets by comparing individuals from different universities to create a competitive context. Considering that people tend to prefer members of their in-group and have a bias toward the members of an out-group [57], future studies could further explore the outcome processing of social comparison with out-group members. In addition, the participants recruited for this study were all college-aged students. Previous studies have shown that children as early as preschool age evaluate themselves through social comparison information [58]. Therefore, it is of great practical significance to explore the neural basis of social comparison outcome evaluation in individuals at different developmental stages.

## 5. Conclusions

In this study, we examined outcome processing in the social comparison context from the perspective of the deviation degree between the self and others. At the ERP level, the effect of the deviation degree of the self versus others in the social comparison was reflected in the FRN and P300 components. Importantly, this progressive effect with the degree of deviation occurred only when participants received positive outcomes rather than negative outcomes. These results suggest that people may develop an empathic response to others when they receive positive outcomes. In contrast, people tend to avoid deeper social comparison that threatens their self-esteem when they receive negative outcomes. Generally, our findings extend the knowledge about outcome processing in social comparison.

## Data Availability

The datasets generated and/or analyzed for the current study are available from the corresponding author on reasonable request.
